# Comparative Evaluation of Sulfur Compounds Contents and Antiobesity Properties of* Allium hookeri* Prepared by Different Drying Methods

**DOI:** 10.1155/2017/2436927

**Published:** 2017-03-16

**Authors:** Min Hye Yang, Na-Hyun Kim, Jeong-Doo Heo, Jung-Rae Rho, Kwang Ju Ock, Eui-Cheol Shin, Eun Ju Jeong

**Affiliations:** ^1^College of Pharmacy, Pusan National University, Busan 46241, Republic of Korea; ^2^Gyeongnam Department of Environment & Toxicology, Korea Institute of Toxicology, 17 Jegok-gil, Munsan-eup, Jinju 52834, Republic of Korea; ^3^Department of Oceanography, Kunsan National University, Jeonbuk 54150, Republic of Korea; ^4^Max Bright Samchae, Jinju Industry Foundation, Munsan-eup, Jinju 52839, Republic of Korea; ^5^Department of Food Science, Gyeongnam National University of Science and Technology, Jinju 52725, Republic of Korea; ^6^Department of Agronomy and Medicinal Plant Resources, Gyeongnam National University of Science and Technology, Jinju 52725, Republic of Korea

## Abstract

Despite the nutritional and medicinal values of* Allium hookeri*, its unique flavor (onion or garlic taste and smell) coming from sulfur containing compounds limits its usage as functional food. For comparative study,* A. hookeri* roots were prepared under two different drying conditions, namely, low-temperature drying that minimizes the volatilization of sulfur components and hot-air drying that minimizes the garlic odor and spicy taste of* A. hookeri*. In GC/MS olfactory system, the odorous chemicals and organosulfur compounds such as diallyl trisulfide, dimethyl trisulfide, and dipropyl trisulfide were significantly decreased in hot-air drying compared to low-temperature drying. The spiciness and saltiness taste were noticeably reduced, while sourness, sweetness, and umami taste were significantly increased in hot-air dried* A. hookeri* according to electronic tongue. Although the content of volatile sulfur components was present at lower level, the administration of hot-air dried* A. hookeri* extract (100 mg/kg* p.o.*) apparently prevented the body weight gain and improved insulin resistance in C57BL/6J obese mice receiving high fat diet. Results suggested that the hot-air dried* A. hookeri* possessing better taste and odor might be available as functional crop and bioactive diet supplement for the prevention and/or treatment of obesity.

## 1. Introduction

The species of* Allium* including* Allium cepa *(onions),* Allium sativum* (garlic), and* Allium schoenoprasum* (chives) have been consumed as medicinal food for a long time [1]. Phytochemically,* Allium* plants contain sulfur compounds as main bioactive constituents [2]. The unique taste and odor of* Allium* species comes from the enzymatic hydrolysis of several precursors of sulfur-containing flavor compounds [3]. Allicin (diallyl thiosulfinate) is a main allyl sulfur component of garlic (*Allium sativum* Linn.) that constitutes over 60% of total garlic thiosulfinates. Thiosulfinate including allicin is a group of very unstable compounds. Once it is generated, it readily gives rise to further rearrangement leading to the production of a variety of derivatives. It has been reported that the amount of organosulfur compounds in the genus* Allium* can be changed depending on the drying conditions such as temperature, time, or an apparatus used [4, 5].

*Allium hookeri* Thwaites (Liliaceae) is a terrestrial perennial herb and widely grown as medicinal food in Southeast Asia countries [6]. The root of* A. Hookeri*, also called “juumyit” or “napakpi,” has been traditionally used to treat inflammatory diseases in India and Myanmar [7]. The* A. hookeri* is known to have biological properties such as antioxidant, anticancer, and anti-inflammatory effects [7–9]. The therapeutic benefits of* A. hookeri* are attributed to the presence of diverse phenols, phytosterols, and organosulfur compounds [10].* A. hookeri* contains a strong flavor which comes from the high content of diallyl sulfide, a flavor component of garlic* (A. sativum)*. It has been reported that diallyl sulfide is contained in the* A. hookeri* with a higher level of abundance than in the* A. sativum* [9].

Obesity arises from dysregulation of energy balance due to excessive energy intake and insufficient energy expenditure [11]. Epidemic increase in overweight and obesity is of medical concern because it is an important risk factor for several chronic diseases, particularly dyslipidemia and type 2 diabetes [12, 13]. Excessive body weight gain has been intimately associated with insulin resistance in individuals with type 2 diabetes [14]. Besides, obesity is a strong causal factor for sleep-disordered breathing, which contributes to the increased cardiovascular mortality [15]. Naturally occurring products have attracted researchers' attentions as sources of new drugs and drug leads for the treatment of obesity [16–18]. The species of* Allium* have been used as a folk medicine for the treatment of metabolic diseases and* Allium*-derived extracts have recently been of interest for their antiobesity effects [19, 20]. However there has not been any attempt to reveal the beneficial effects of* A. hookeri* in prevention and/or treatment of obesity.

Despite important therapeutic and culinary value, people avoid the* A. hookeri* because of their unique odor and spicy taste induced by volatile sulfur compounds [21]. Therefore, we attempted to develop the optimized drying methods of* A. hookeri* that minimize the unpleasant odor and taste without reducing its biological activity. Two dried* A. hookeri* have been prepared under the different drying operation, namely, low-temperature drying and hot-air drying. The contents of organosulfur, odorous components, were compared, and sensory test has been performed. Also, in vivo test was undertaken to ascertain the antiobesity effect of two types of* A. hookeri* in high-fat diet-induced C57BL/6J obese mouse model.

## 2. Materials and Methods

### 2.1. Plant Material and Reagents

*A. hookeri* was cultivated and harvested in Hadong (Korea). The roots of* A. hookeri* were washed three times with five volumes of distilled water. A voucher specimen was deposited in Laboratory of Pharmacognosy in Gyeongnam National University of Science and Technology.

### 2.2. Drying Procedures and Sample Preparation

*A. hookeri* roots were dried for 96 h in an oven at 40°C to minimize the volatilization of sulfur compounds contained. The darkened* A. hookeri* roots were autoclaved for 10 min at 105°C, then steamed for 30 min at 80°C, and further steamed for 24 h at 70°C followed by drying for 72 h at 35°C. A Likens and Nickerson-type simultaneous steam distillation and extraction apparatus (SDE) was used for the extraction of volatile compounds. Ground samples (100 g) were mixed with 1 L of distilled water followed by the addition of internal standard (1 mL of pentadecane, 1 mg/mL, Sigma-Aldrich Co.). Atmospheric steam distillation was performed to collect volatile oils from the sample in a 100 mL mixture of pentane and diethyl ether (1 : 1, v/v) at 110°C for 3 h. Anhydrous sodium sulfate (10 g) was added to the extract, which was then maintained at 4°C overnight. Samples were then filtered and reduced to a volume of 1 mL using a nitrogen evaporator.

### 2.3. Gas Chromatography-Mass Spectrometry (GC-MS) Analysis

The essential oil was analyzed using an Agilent 7890A and 5975C (Agilent Technologies) and HP-5MS capillary column (30 m × 0.25 mm × 0.25 *μ*m film thickness). Injector and detector temperatures were set at 220°C and 290°C, respectively. Column temperature was initially kept at 40°C for 5 min and then gradually increased to 200°C at a rate of 5°C/min. Helium was used as carrier gas at a flow rate of 1 mL/min. Samples of 1 *μ*L were injected manually in the splitless mode. Tentative identification of the compounds was based on the comparison of their mass spectra with those of NIST 98 and Wiley 275 library data of the GC/MS system. Quantitative data were obtained electronically from FID area percent data without the use of correction factors.

### 2.4. Gas Chromatography-Olfactometry

In parallel with GC-MS, samples were analyzed by GC/MS-olfactory detection port with heated mixing chamber (ODP 3, Gerstel, Inc., Linthicum, MD, USA) using the concept of detection frequency. A sniffing procedure panel was formed of 3 judges who were chosen from 10 assessors well trained in sensory evaluation. Results of GC/MS-O analyses were expressed as average values of odor intensity in a scale from 1 to 7 with increments of 1, obtained from 3 independent measurements.

### 2.5. Sensory Evaluation Test

The Astree II e-Tongue system, developed by Alpha M.O.S. (Toulouse, France), was used for taste evaluation of the test example solutions. Astree II is a fully automated taste analyzer equipped with seven sensors, ZZ, AB, GA, BB, CA, DA, and JE, based on the ChemFET technology (Chemical Modified Field Effect Transistor) for liquid samples analysis (Alpha M.O.S., 2004). In the presence of dissolved compounds, a potentiometric difference is measured between each of the seven sensors and the Ag/AgCl reference electrode. Each sensor has a specific organic membrane, which interacts with ionic, neutral, and chemical compounds present in the sample solution in a specific manner. Any interaction at the membrane interface is detected by the sensor and converted into an electronic signal. The raw data is expressed as voltage versus time. For these experiments, only the last 20 s of the 120 s data was used in the analysis. Samples were replicated five times and the average value of the last four measurements is used in the data analysis. The sensors were rinsed in water following each analysis to prevent cross-contamination between samples.

### 2.6. Animals and Diets

Male C57BL/6J mice (3 weeks old) were purchased from Central Lab., Animal Inc. (Seoul, Korea). Animals were acclimatized for two weeks under a 12 h : 12 h light-dark cycle and constant temperature (20 ± 2°C) and humidity (50 ± 5%), with water and food freely available. The mice were divided into 4 groups and then fed a normal diet containing 10% kcal fat (3.85 kcal/g) or a high-fat diet containing 60% kcal fat (5.24 kcal/g) (Research Diets Inc., New Brunswick, NJ). EALT dissolved in 0.5% CMC was orally administered (100 mg/kg body weight) once a day for 8 weeks. The high-fat diet mice received either 15 mg/kg body weight Orlistat (Xenical®, Roche Pharma Ltd., Reinach, Switzerland) or 0.5% CMC (10 ml/kg body weight) orally as a positive or negative control. A normal diet group was also treated with only 0.5% CMC (10 ml/kg body weight) as a vehicle. All animal experiments were carried out according to the guidelines of the Gyeongnam Department of Environment & Toxicology, Korea Institute of Toxicology, on the Care and Use of Laboratory Animals. Four groups are as follows.

  Group I (ND) received oral 0.5%-CMC and was fed a normal diet (normal control group).
  Group II (VC) received oral 0.5%-CMC and was fed a high-fat diet, used as a disease group (negative control group).
  Group III (PC) received Orlistat (15 mg/kg body weight/rat, p.o.) and was fed a high-fat diet (positive control group).
  Group IV (T1) received the low-temperature dried* A. hookeri* (100 mg/kg body weight/rat, p.o.) and was fed a high-fat diet.
  Group IV (T2) received hot-air dried* A. hookeri* (100 mg/kg body weight/rat, p.o.) and was fed a high-fat diet.

### 2.7. Measurement of Body and Adipose Tissue Weight, Nonfasting Blood Glucose, and Blood Biochemistry

The body weight and blood glucose concentration were measured under the condition of nonfasting once a week. After 8 weeks of treatment, mice were sacrificed by cervical dislocation and the epididymal and perirenal adipose tissue were dissected and weighed. Nonfasting blood glucose level was measured by a standard method using a glucometer (Accu-Chek Active; Roche Applied Science, Indianapolis, IN). Blood samples were obtained from the abdominal aorta and centrifuged at 1,500 rpm for 15 min to separate plasma and blood cells [22]. The plasma levels of triglyceride (TG), total cholesterol (TC), low-density lipoprotein cholesterol (LDLC), and high-density lipoprotein cholesterol (HDLC) were estimated using assay kits (Asan Pharmaceutical Co., Seoul, Korea).

### 2.8. Determination of Oral Glucose Tolerance Test (OGTT) and Intraperitoneal Insulin Tolerance Test (IPITT)

Nonfasting glucose level was measured once a week during 8 weeks of diet feeding. OGTT and IPITT were performed after 7 weeks on the HFD. Mice weighing over 30 g were selected and fasted for 4 hours followed by glucose (2 g/kg) given orally for OGTT, or insulin (1 U/kg; Actrapid, Novo Nordisk, Bagsvaerd, Denmark) was given intraperitoneally for IPITT, at time 0. Two hundred *μ*l of blood was sampled from tail at 15, 30, 60, and 120 minutes for both OGTT and IPITT. Blood glucose concentration was determined with a glucometer (Accu-Chek Active; Roche Applied Science, Indianapolis, IN).

### 2.9. Statistical Analysis

Each data value was presented as the mean ± SD. Data of body weight were analyzed by two-way ANOVA, while in vitro assay and in vivo biochemical parameters were analyzed by one-way ANOVA. The data were considered to be significant statistically if the probability had a value of 0.05 or less.

## 3. Results

### 3.1. Comparison of Low-Temperature and Hot-Air Drying Methods for the Amount of Volatile Sulfur Compounds from* A. hookeri*

**Figure 1:**
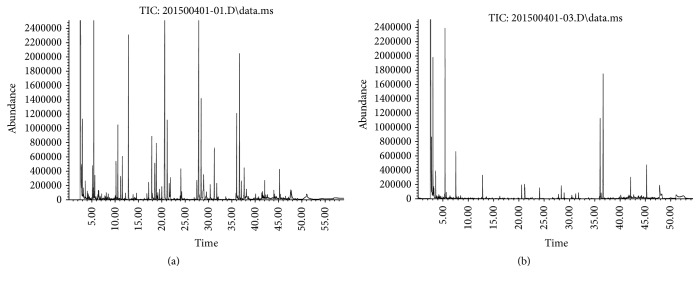
Chromatogram of volatile compounds of low-temperature dried* A. hookeri*, T1 (a), and hot-air dried* A. hookeri*, T2 (b). Labels corresponding to the peaks are given in Table 1.

Figure 1 presented the GC chromatographic profiles of the sulfur-containing volatile compounds in* A. hookeri* dried by low-temperature (Figure 1(a)) or hot-air method (Figure 1(b)). Peak identification for the chromatograms is given in Table 1 along with the organosulfurs content (mg/100 g) of the* A. hookeri* extracts. Major sulfur components contained in* A. hookeri* were identified as diallyl trisulfide, dimethyl trisulfide, dipropyl trisulfide, diallyl disulfide, and methyl allyl disulfide. In detail, diallyl trisulfide was the primary volatile (10.46 mg/100 g) in low-temperature-dried* A. hookeri* (T1), followed by dimethyl trisulfide (6.30 mg/100 g), dipropyl trisulfide (2.74 mg/100 g), and diallyl disulfide and methyl allyl disulfide (2.53 mg/100 g). The main organosulfur compounds were detected in decreased abundance in hot-air/long-time dried* A. hookeri* (T2) compared to T1. Low thermal treatment decreased the content of major sulfide compounds from T2, diallyl trisulfide (98.09% decrease), dimethyl trisulfide (85.56% decrease), dipropyl trisulfide (90.15% decrease), diallyl disulfide (100% decrease), and methyl allyl disulfide (98.81% decrease).

### 3.2. Effects of T1 and T2 in GC-MS-Olfactory and Sensory Tests

**Figure 2:**
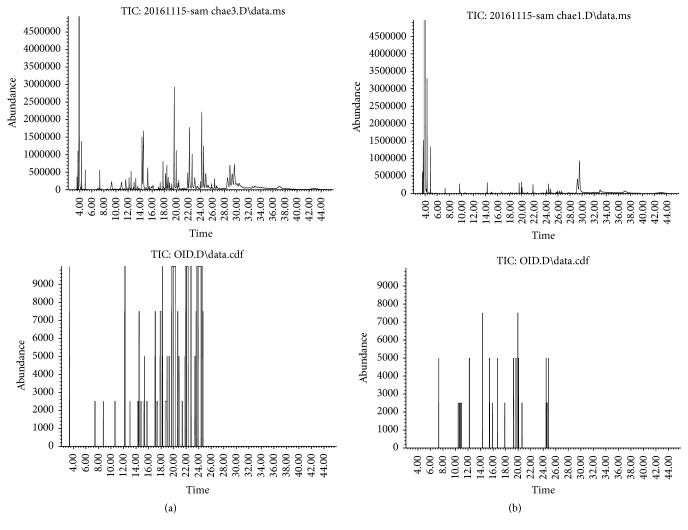
Chromatogram of volatile compounds and aromagram by the sniffing test of T1 (a) and T2 (b). Labels corresponding to the peaks in aromagram are given in Tables 2 and 3.

**Figure 3:**
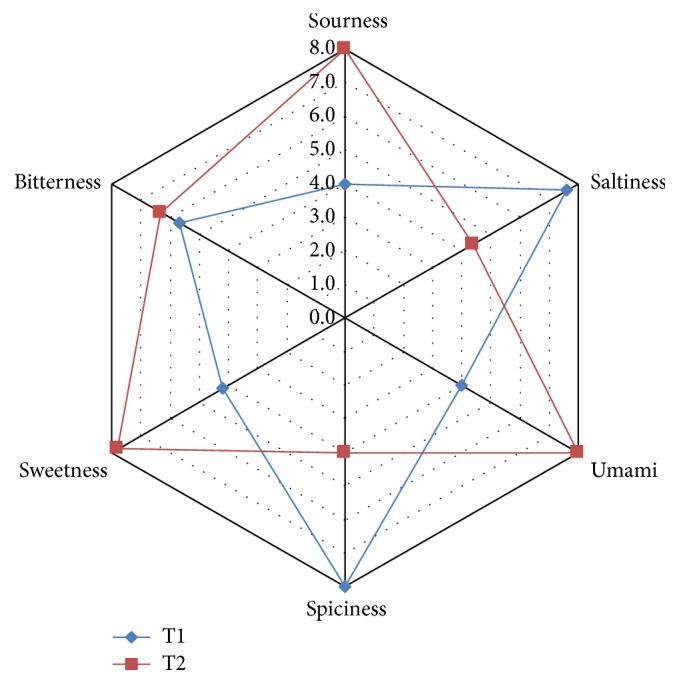
Consumer acceptance of T1 and T2. Blue line represents T1 (low-temperature dried* A. hookeri*) and red line represents T2 (hot-air dried* A. hookeri*).

Principal information on the odor profile of T1 and T2 was obtained by utilizing a GC-MS-Sniff technique. As shown in Figure 2, the intensity of the olfactory peaks in T1 was markedly changed by hot-air and long-time thermal drying in T2. In comparison to T1 (Figure 2(a) and Table 2), most of the sulfur-containing compounds were detected at lower intensities in T2 (Figure 2(b) and Table 3). It was found that some important odorous sulfides such as di-2-propenyl trisulfide and dimethyl tetrasulfide were almost removed (>95%) in the aromagram of T2. The taste evaluation of T1 and T2 was performed with the electronic tongue data with six sensors including salty, sour, sweet, bitter, spicy, and umami (Figure 3). The spiciness and saltiness scores of T2 decreased to 42.11% and 50%, respectively, when compared to the T1 sample. In contrast, the sensory scores of sourness, sweetness, and umami taste of T2 were 100%, 100%, and 85.71% higher than that of T1. The difference in bitter taste between the treatment groups was not statistically significant.

### 3.3. Effects of T1 and T2 on Body Weight Gain, Adipose Tissue Weight, Nonfasting Blood Glucose Level, and Blood Chemistry in High-Fat Diet-Induced Mice

**Figure 4:**
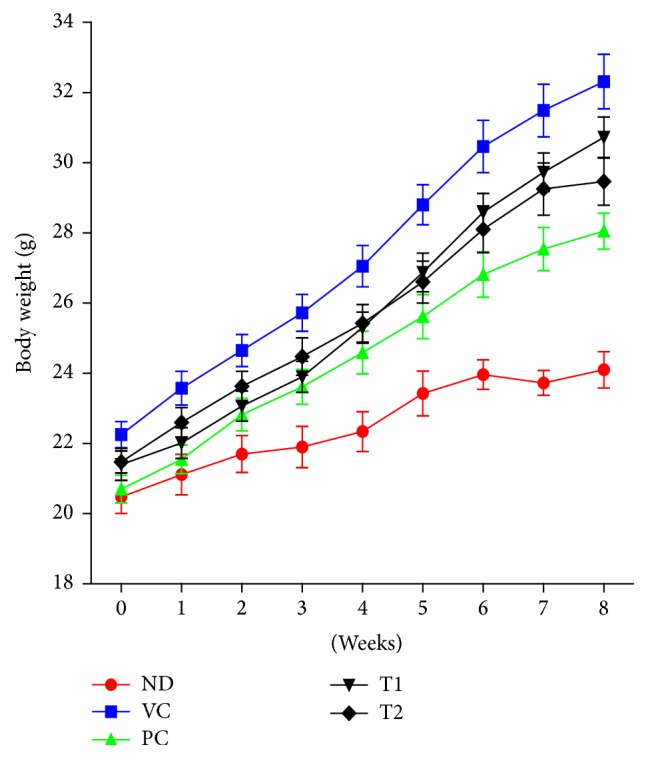
Effects of T1 and T2 on body weight of obese mice fed a high-fat diet for 8 weeks. Values were expressed as the means ± SD (*n* = 10). ND, normal diet group; VC, high-fat diet group; PC, Orlistat-treated group; T1, low-temperature dried* A. hookeri*-treated group; T2, hot-air dried* A. hookeri*-treated group.

**Figure 5:**
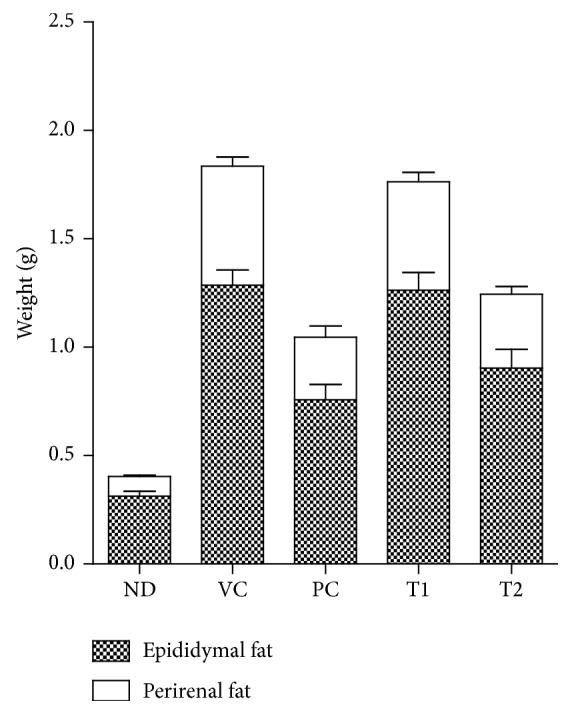
Effect of T1 and T2 on adipose tissue weights in high-fat diet-induced obese mice. Values were expressed as the means ± SD (*n* = 10). ND, normal diet group; VC, high-fat diet group; PC, Orlistat-treated group; T1, low-temperature dried* A. hookeri*-treated group; T2, hot-air dried* A. hookeri*-treated group.

**Figure 6:**
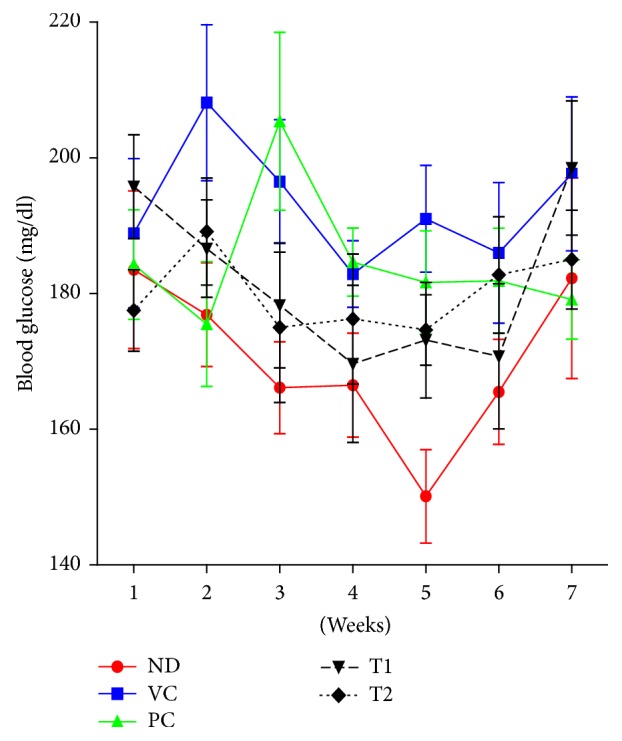
Effect of T1 and T2 on nonfasting blood glucose. Values were expressed as the means ± SD (*n* = 10). ND, normal diet group; VC, high-fat diet group; PC, Orlistat-treated group; T1, low-temperature dried* A. hookeri*-treated group; T2, hot-air dried* A. hookeri*-treated group.

The body weight gain of mice fed a high-fat diet was greater than the value for the ND (normal diet) group (Figure 4). A 2.8-fold increase in body weight gain was observed in VC (high-fat diet + vehicle control) group compared with ND group. Orlistat is an antiobesity drug that is currently available as a strong gastrointestinal lipase inhibitor [23]. In the present study, Orlistat has been used as a positive control in which it effectively decreased weight gain by 26.96% (*P* < 0.01) at the end of experiment (week 8). The average body weights of T1- and T2-administered mice (30.73 g and 29.46 g, resp.) were considerably lower than that of the VC group (32.31 g). The adipose tissue weight, including epididymal and perirenal fat, of obese mice was also markedly reduced by treatment of Orlistat and* A. hookeri* (Figure 5). Cumulative fat mass of high-fat diet group was estimated as the 4.5-fold value of nontreated mice. Treatment of Orlistat and T2 lowered the adipose tissue weight to 42.98% and 32.15%, respectively, when compared to the VC group. Nonfasting blood glucose concentrations of the five groups of mice were shown in Figure 6. High-fat diet feeding (197.63 mg/dl) increased in the nonfasting blood glucose level compared to the ND group (182.25 mg/dl) after seven weeks. T2 administration markedly prevented the elevation of blood glucose by 82.11% compared to VC group. However, the difference in blood glucose level between the T1 treatment group and VC group was not statistically significant. The remarkable change in blood biochemistry was the reduction in the level of triglyceride in* A. hookeri*-treated groups. The increased level of triglyceride in VC (127.06 mg) compared to ND (85.38 mg) was significantly decreased by the treatment with T1 (51.76 mg) or T2 (45.92 mg) that was more potent than positive control, Orlistat-treated group (65.98 mg) (Table 4).

### 3.4. Effects of T1 and T2 on High-Fat Diet-Induced Glucose Intolerance and Insulin Resistance

**Figure 7:**
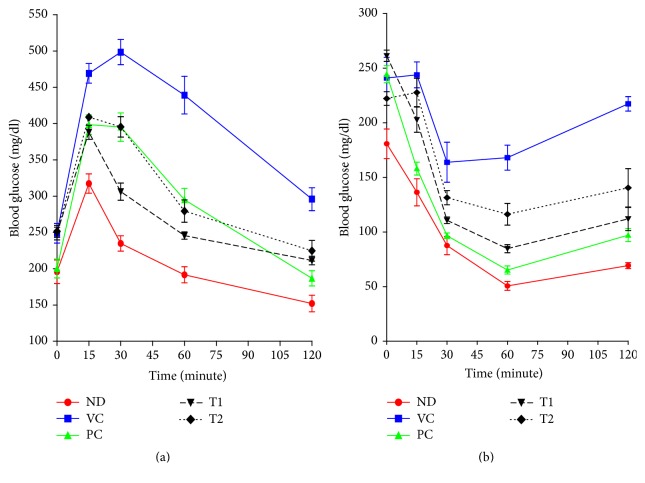
Effect of EALT on OGTT (a) and IPITT (b). Values were expressed as the means ± SD (*n* = 10). ND, normal diet group; VC, high-fat diet group; PC, Orlistat-treated group; T1, low-temperature dried* A. hookeri*-treated group; T2, hot-air dried* A. hookeri*-treated group.

OGTT and IPITT were performed to evaluate the effect of* A. hookeri* on glucose and insulin tolerance. Feeding a high-fat diet to mice for 8 weeks developed severe glucose intolerance and insulin resistance, characterized by increased area under the blood glucose curves (Figure 7). During OGTT, the blood glucose of high-fat diet-fed mice increased to 498.38 mg/dl after 30 min from a baseline value of 248.75 mg/dl (Figure 7(a)). The blood glucose levels of T1 and T2 group decreased to 306.25 mg/dl and 395.38 mg/dl, respectively, at 30 min. In IPITT, mice treated with a high-fat diet also exhibited severe insulin resistance (Figure 7(b)). In mice treated with T1 and T2 following i.p. insulin load, the level of blood glucose decreased to 110.75 mg/dl and 131.50 mg/dl, respectively, after 30 min in comparison to the vehicle control (163.88 mg/dl). Blood glucose levels of PC, a positive control, were 395.13 for OGTT and 96.63 for IPITT after 30 min, which were 20.72% and 41.04% less as compared to corresponding values observed in high-fat diet control mice.

## 4. Discussion

Drying is an ancient process used to preserve botanical medicines. Drying process is based on the dehydration of plants to extend the shelf-life and improve the nutritional value [22, 24]. Various drying techniques, hot-air, microwave, and freeze drying, and operating conditions have been applied in the preparation of botanical samples [25, 26]. Herbal extracts might vary depending on different operating conditions including temperature, relative humidity, and air velocity even in the same drying process [27]. Drying has significant effects on the production of quality medicinal plants. In addition, the constituent profiles of plants can be affected by the choice of drying method and thereby adoption of proper drying technique is important [26].

To compare the relative composition of volatile sulfur compounds,* A. hookeri* samples were prepared by two different drying methods, conventional low-temperature drying system minimizing the loss of volatile sulfur compounds and the optimized hot-air/long-time drying system minimizing unpleasant odor and taste. The characteristic flavor of* Allium* species mainly consists of odorous sulfur compounds [28]. The amount of main sulfur constituents (diallyl trisulfide, dimethyl trisulfide, dipropyl trisulfide, diallyl disulfide, and methyl allyl) was significantly lower in* A. hookeri* dried with hot-air (T2) than in* A. hookeri* dried at low-temperature (T1). Furthermore, some sulfur compounds of T1 such as diallyl disulfide, dimethyl tetrasulfide, (*E*,*E*)-bis(1-propenyl) disulfide, and methyl-trans-propenyl-disulfide did not exist in T2 sample. According to the previous literatures, high temperature convective drying normally resulted in poor sulfur compounds retention and moderate air temperatures allowed a better sulfur compounds content than harsh thermal treatment [5, 6]. Consistent with the report, in our present study, drastic qualitative and quantitative reduction of the major sulfide compounds was observed after hot-air thermal drying.

The GC/MS data of T1 and T2 were correlated with the result of their GC/MS-olfactometry. Comparison of the chromatographic odor profiles between T1 and T2 showed that the odor intensity of most of the olfactory peaks was definitely weakened in T2. To verify the reduced spiciness of T2, actual score of spicy taste was determined by human sensory testing using an electronic tongue. In comparison to the T1, T2 treatment revealed a significant decrease in spiciness and saltiness but increase in sweetness and umami taste. Spicy odor and flavor of* Allium* plants are known to be occur as a result of generation of major sensory-active sulfur compounds [29]. In general, the tastes of sweet and umami cause food acceptance behavior, whereas the bitter and salty tastes elicit avoidance [30]. According to overall GC/MS-olfactometry and sensory test results, T2 exhibited better removal of taste- and odor-causing compounds and thereby the optimized process of drying with hot-air might encourage acceptance and consumption of* A. hookeri* products.

Antiobesity and hypolipidemic effects of* A. hookeri* were investigated in dietary obese mice. It has been reported that animal model of obesity induced by high-fat diet resembles the human obesity [31]. Adipocyte hypertrophy, hyperplasia, and insulin resistance, similar to those in human obese subjects, were observed in rodents after high-fat diet feeding [31]. In our present study, as expected, exposure of mice to a high-fat diet caused severe obesity characterized by increased body weight and adiposity. A significant reduction in body weight gain and fat deposition in obese mice was detected by treatment of* A. hookeri*. Particularly, the antiobesity effect was more pronounced with T2 in comparison to T1. According to histological analysis of epididymal fat, adipocytes from VC group were markedly larger than those from ND group. An increase in total white adipose tissue mass of epididymal and perirenal fat under high-fat diet was inhibited in 100 mg/kg T2-administered mice. Sulfur-containing compounds, such as allicin and allyl methyl sulfide, are known to be the main responsible constituents of* Allium* plants for most of their pharmacological effects [32, 33]. Although total amount of volatile sulfur compounds tended to be lower in T2 than in T1, T2 showed better activity for decreasing weight gain and adiposity in high-fat diet-induced animal model.

Consumption of high levels of dietary fat is considered to be a major factor in the promotion of hyperglycemia and whole-body insulin resistance [34]. An elevated glucose concentration is a common feature of obesity itself or closely linked metabolic diseases [31]. In this regard, high-fat diet-induced obesity is directly connected with an increased risk of type 2 diabetes [35]. Our observations of hyperglycemia and insulin tolerance in mice fed high-fat diet confirmed induction of dietary obesity. The increased level of nonfasting blood glucose induced by high-fat diet was attenuated by T2 treatment for seven weeks. Besides, T2 coadministration with high-fat diet prevented both glucose and insulin tolerance during OGTT and IPITT. Taken together, T2 might contribute to improvement of insulin sensitivity and resultant lowering of the elevated blood glucose level in obesity in spite of its reduction of sulfur-containing components.

## 5. Conclusion

Collectively, the content of major sulfide compounds was noticeably diminished by the optimized thermal drying with hot-air and thereby a remarkable decrease in spiciness and odorous intensities was observed in T2. Administration of T2 effectively prevented weight gain and decreased body fat mass induced by high-fat diet feeding in animal model, although bioactive sulfur-containing compounds were decreased in T2. T2 was also useful in improving blood chemistry and mitigating both glucose tolerance and insulin tolerance in dietary obese mice. Further study is warranted to explore the active components responsible for the antiobesity effect of T2.

## Figures and Tables

**Table 1:** Sulfur-containing volatile compounds in samples by GC/MS.

RT (min)^1^	Compounds	Content (mg/100 g)	
		T1	T2
4.27	Ethyl sulfide	—^2^	0.07
5.14	Dimethyl sulfide	1.18	0.06
8.37	3,3-Thiobis-1-propene	0.07	—
8.90	n-Propyl cis-1-propenyl sulfide	0.06	—
10.58	Methyl allyl disulfide	2.53	0.03
10.82	Diethyl disulfide	—	0.02
11.17	Methyl-trans-propenyl-disulfide	1.32	—
11.22	Methyl propyl disulfide	—	0.04

11.55	Trans-propenyl methyl disulfide	—	0.01
12.58	Dimethyl trisulfide	6.30	0.91
17.17	Propene sulfide	0.70	—
17.85	Diallyl disulfide	2.53	—
18.28	Butyl propenyl sulfide	—	—
18.83	(E,E)-Bis(1-propenyl) disulfide	2.33	—
19.18	trans-Propenyl propyl disulfide	—	0.03
19.51	2-Propenyl propyl disulfide	0.45	0.01
20.60	Methyl methylthiomethyl disulfide	—	—
21.14	Methyl propyl trisulfide	—	0.62
24.09	Dimethyl tetrasulfide	1.32	—
27.91	Diallyl trisulfide	10.46	0.20
28.94	Dipropyl trisulfide	2.74	0.27
31.25	Methyl 2-propenyl tetrasulfide	—	0.23
34.39	Dimethyl trisulfide	—	0.04
37.65	Di-2-propenyl tetrasulfide	1.51	0.05

^1^RT: retention time. ^2^“—” corresponds to “not detected.”

**Table 2:** Odorous compounds in T1 by GC/MS-olfactometry.

RT (min)^1^	Compounds	Content (ug/100 g)
6.93	Dimethyl disulfide	0.11
8.49	2,3,3-Trimethyl-1,4-pentadiene	0.02
8.61	Hexanal	0.07
9.66	5-Methyl-2-thiophenecarboxaldehyde	0.14
9.97	6,6-Dimethylhepta-2,4-diene	0.09
10.16	1,6-Dimethyl-1,5-cyclooctadiene	0.07
10.27	1,2,4,4-Tetramethylcyclopentene	0.07
10.67	Ethyl-benzene	0.17
11.63	1,2-Bis(allyl)disulfane	2.73
12.17	3-(Methylthio)-propanal	1.72
13.00	Methyl propyl disulfide	1.06
13.63	Methyl (methylthio)methyl disulfide	0.90
14.32	Dimethyl trisulfide	5.22
14.98	2-Pentyl-furan	0.24
15.51	Cyclodecanone	0.34
16.70	Benzeneacetaldehyde	0.11
17.02	Dodecane	0.27
17.19	2-Formyl-3-methylthiophene	0.09
17.90	Di-2-propenyl disulfide	0.34
18.44	Dimethyl tetrasulfide	10.81
20.42	1,2-Dithiane-4,5-diol	1.38
21.78	3-Vinyl-1,2-dithiacyclohex-5-ene	0.15
22.19	Di-2-propenyl trisulfide	13.73
22.62	2,4-Dihydro-4-methyl-3H-1,2,4-triazole-3-thione	6.91
22.80	1-Ethoxyvinyl chloroacetate	0.27
24.22	Di-2-propenyl trisulfide	13.94
24.53	2,4-Dihydro-4-methyl-3H-1,2,4-triazole-3-thione	7.04
26.34	Methyl 2-propenyl disulfide	1.97

^1^RT: retention time.

**Table 3:** Odorous compounds in T2 by GC/MS-olfactometry.

RT (min)^1^	Compounds	Content (ug/100 g)
9.72	2-Furancarboxaldehyde	1.05
10.55	2-Furanmethanol	0.08
11.73	Pentanoic acid	0.05
12.38	1-(2-Furanyl)-ethanone	0.05
13.00	Methyl propyl disulfide	0.04
14.11	5-Methyl-2-furancarboxaldehyde	0.09
14.29	Dimethyl trisulfide	1.03
15.71	Hexanoic acid	0.09
16.68	1H-Pyrrole-2-carboxaldehyde	0.17
16.79	Benzeneacetaldehyde	0.02
17.01	Dihydro-*p*-tolualdehyde	0.05
17.80	Heptanoic acid	0.06
18.36	2,6,11,15-Tetramethyl-hexadecane	0.02
18.46	Nonanal	0.05
18.88	Phenylethyl alcohol	0.07
19.08	2-Ethyl-hexanoic acid	0.13
20.63	Octanoic acid	0.13
21.87	Dimethyl tetrasulfide	1.03
23.39	Tetradecane	0.08
24.12	Di-2-propenyl trisulfide	0.44
24.80	Dipropyl trisulfide	0.65
26.29	Methyl 2-propenyl disulfide	0.40

^1^RT: retention time.

**Table 4:** Blood biochemistry in high-fat diet-induced obese mice.

	HDLC^1^	LDLC^2^	TC^3^	TG^4^
ND	62.54	5.84	95.80	85.38
VC	81.74	5.70	135.00	127.06
PC	75.34	8.44	130.00	65.98
T1	81.24	7.02	142.80	51.76
T2	79.04	7.38	142.00	45.92

^1^HDLC: high-density lipoprotein cholesterol.

^2^LDLC: low-density lipoprotein cholesterol.

^3^TC: total cholesterol.

^4^TG: triglyceride.

## References

[1] Block E. (1985). The chemistry of garlic and onions. *Scientific American*.

[2] Lanzotti V. (2006). The analysis of onion and garlic. *Journal of Chromatography A*.

[3] Schutte L. (1974). Precursors of sulfur-containing flavor compounds. *C R C Critical Reviews in Food Technology*.

[4] Aware R. S., Thorat B. N. (2011). Garlic under various drying study and its impact on allicin retention. *Drying Technology*.

[5] Ratti C., Araya-Farias M., Mendez-Lagunas L., Makhlouf J. (2007). Drying of garlic (*Allium sativum*) and Its effect on allicin retention. *Drying Technology*.

[6] Ayam V. (2011). *Allium hookeri*, Thw. Enum. A lesser known terrestrial perennial herb used as food and its ethnobotanical relevance in Manipur. *African Journal of Food, Agriculture, Nutrition and Development*.

[7] Bae G. C., Bae D. Y. (2012). The anti-inflammatory effects of ethanol extract of *Allium Hookeri* cultivated in South Korea. *The Korea Journal of Herbology*.

[8] Lee K. W., Kim Y. S., Park P. J., Jeong J. H. (2014). Comparison of effect of water and ethanolic extract from roots and leaves of *Allium hookeri*. *Journal of the Korean Society of Food Science and Nutrition*.

[9] Kim C. H., Lee M. A., Kim T. W., Jang J. Y., Kim H. J. (2012). Anti-inflammatory effect of *Allium hookeri* root methanol extract in LPS-induced RAW264.7 cells. *Journal of the Korean Society of Food Science and Nutrition*.

[10] Song E. Y., Pyun C. W., Hong G. E., Lim K. W., Lee C. H. (2014). Effect of addition of *Allium hookeri* on the quality of fermented sausage with meat from sulfur fed pigs during ripening. *Korean Journal for Food Science of Animal Resources*.

[11] Rössner S. (2002). Obesity: the disease of the twenty-first century. *International Journal of Obesity and Related Metabolic Disorders*.

[12] Flegal K. M., Graubard B. I., Williamson D. F. (2007). Cause-specific excess deaths associated with underweight, overweight, and obesity. *Obstetrical & Gynecological Survey*.

[13] Nikolopoulou A., Kadoglou N. P. (2012). Obesity and metabolic syndrome as related to cardiovascular disease. *Expert Review of Cardiovascular Therapy*.

[14] Bonadonna R. C., Groop L., Kraemer N., Ferrannini E., Prato S. D., DeFronzo R. A. (1990). Obesity and insulin resistance in humans: a dose-response study. *Metabolism*.

[15] Wolk R., Shamsuzzaman A. S., Somers V. K. (2003). Obesity, sleep apnea, and hypertension. *Hypertension*.

[16] Newman D. J., Cragg G. M. (2007). Natural products as sources of new drugs over the last 25 years. *Journal of Natural Products*.

[17] Sergent T., Vanderstraeten J., Winand J., Beguin P., Schneider Y.-J. (2012). Phenolic compounds and plant extracts as potential natural anti-obesity substances. *Food Chemistry*.

[18] Yun J. W. (2010). Possible anti-obesity therapeutics from nature—a review. *Phytochemistry*.

[19] Kim Y., Lee M. S., Kim J. S. (2007). Garlic decreases body weight via decrease of serum lipid and increase of uncoupling proteins mRNA expression. *The FASEB Journal*.

[20] Joo H., Kim C. T., Kim I. H., Kim Y. (2013). Anti-obesity effects of hot water extract and high hydrostatic pressure extract of garlic in rats fed a high-fat diet. *Food and Chemical Toxicology*.

[21] Havey M. J., Janick J. (1999). Advances in new Alliums. *Perspectives on New Crops and New Uses*.

[22] Ratti C. (2001). Hot air and freeze-drying of high-value foods: a review. *Journal of Food Engineering*.

[23] Heck A. M., Yanovski J. A., Calis K. A. (2000). Orlistat, a new lipase inhibitor for the management of obesity. *Pharmacotherapy*.

[24] Orsat V., Vijaya Raghavan G. S., Sosle V. (2008). Adapting drying technologies for agri-food market development in India. *Drying Technology*.

[25] Mahanom H., Azizah A. H., Dzulkifly M. H. (1999). Effect of different drying methods on concentrations of several phytochemicals in herbal preparation of 8 medicinal plants leaves. *Malaysian Journal of Nutrition*.

[26] Abascal K., Ganora L., Yarnell E. (2005). The effect of freeze-drying and its implications for botanical medicine: a review. *Phytotherapy Research*.

[27] Ong E. S. (2004). Extraction methods and chemical standardization of botanicals and herbal preparations. *Journal of Chromatography B*.

[28] Boelens M., De Valois P. J., Wobben H. J. (1971). Volatile flavor compounds from onion. *Journal of Agricultural and Food Chemistry*.

[29] Li X., Staszewski L., Xu H., Durick K., Zoller M., Adler E. (2002). Human receptors for sweet and umami taste. *Proceedings of the National Academy of Sciences of the United States of America*.

[30] Scott K. (2005). Taste recognition: food for thought. *Neuron*.

[31] Buettner R., Schölmerich J., Bollheimer L. C. (2007). High-fat diets: modeling the metabolic disorders of human obesity in rodents. *Obesity*.

[32] Mikaili P., Maadirad S., Moloudizargari M., Aghajanshakeri S., Sarahroodi S. (2013). Therapeutic uses and pharmacological properties of garlic, shallot, and their biologically active compounds. *Iranian Journal of Basic Medical Sciences*.

[33] Ban J. O., Lee D. H., Kim E. J. (2012). Antiobesity effects of a sulfur compound thiacremonone mediated via down-regulation of serum triglyceride and glucose levels and lipid accumulation in the liver of db/db mice. *Phytotherapy Research*.

[34] Rivellese A. A., De Natale C., Lilli S. (2002). Type of dietary fat and insulin resistance. *Annals of the New York Academy of Sciences*.

[35] Kahn B. B., Flier J. S. (2000). Obesity and insulin resistance. *Journal of Clinical Investigation*.

